# In-Hospital Mortality among Ischemic Stroke Patients in Gondar University Hospital: A Retrospective Cohort Study

**DOI:** 10.1155/2019/7275063

**Published:** 2019-01-01

**Authors:** Eyob Alemayehu Gebreyohannes, Akshaya Srikanth Bhagavathula, Tamrat Befekadu Abebe, Mohammed Assen Seid, Kaleab Taye Haile

**Affiliations:** ^1^Department of Clinical Pharmacy, University of Gondar, Gondar, Ethiopia; ^2^Department of Internal Medicine, College of Medicine and Health Sciences, UAE Univerisity, Abu Dhabi, UAE; ^3^Department of Learning, Informatics, Management, and Ethics, Karolinska Institutet, Solna, Sweden; ^4^Department of Pharmaceutics, University of Gondar, Gondar, Ethiopia

## Abstract

**Introduction:**

Ischemic stroke is the third leading cause of mortality in low-income countries and the sixth in Ethiopia. The aim of this study was to determine the rate and predictors of in-hospital mortality due to ischemic stroke in Gondar University Hospital.

**Methods:**

The study was conducted from April 1, 2017, to May 15, 2017, at Gondar University Hospital. A census using retrospective cohort study design was conducted on medical records of adult patients with the diagnosis of ischemic stroke attending the medical inpatient ward of Gondar University Hospital between November 2012 and September 2016. Cox hazard regression was used to determine the predictors of in-hospital mortality. A two-sided statistical test at 5% level of significance was used.

**Results:**

The mean (±SD) duration of hospital stay was 11.55 (10.040) days. Of the total 208 patients, 26 (12.5%) patients died in the hospital. Cox regression revealed that only a decrease in renal function, particularly elevated serum creatinine (AHR=8.848, 95% CI: 1.616-67.437), was associated with a statistically significant increase of in-hospital mortality. The symptom onset-to-admission time varied greatly among patients and ranged from 1 hour to 168 hours.

**Conclusion:**

The in-hospital mortality associated with ischemic stroke was found to be high. Mainly, elevation in serum creatinine was highly associated with poorer outcomes in terms of in-hospital mortality. Much work should be done on improving the knowledge and awareness of the community regarding ischemic stroke and stroke in general to encourage early medical seeking behavior and reduce mortality and long-term disability.

## 1. Introduction

Stroke is a disease most commonly characterized by sudden weakness or numbness of the face, arm, or leg, most often on one side of the body. It results from inadequate supply of oxygen and nutrients as a result of disruption of blood supply and damage to the brain tissue [1]. The global burden of stroke has been increasing over the years. However, it has shown two trends over the past four decades between high-income and low-income countries. While the incidence of stroke showed a 42% reduction in high-income countries, it showed a greater than 100% increase in low-income countries [2]. Community-based studies reported age-standardized incidence and prevalence rates of stroke in Africa reach up to 316 and 981 per 100 000 population, respectively [3]. While it is now known that the incidence of stroke is higher in developing countries than in developed countries, the factors responsible for such huge differences remain unclear [3, 4].

Stroke is the second most common cause of mortality globally and the third leading cause of mortality in low-income countries [5]. Mortality associated with stroke varies from country to country. More than a tenfold variation has been reported in the age- and sex-adjusted mortality rates between different countries. However, developing countries are bearing the higher burden [6]. Despite lack of high quality data in developing countries, more than two-third deaths associated with stroke occur in developing countries [6, 7].

While stroke can be subdivided into ischemic and hemorrhagic, the relative prevalence of either of the two can vary across the world. In Ethiopia, ischemic stroke has been identified as the sixth leading cause of death [8]. There are only few studies [9, 10] that assessed stroke outcomes in Ethiopia. These studies reported that the incidence of ischemic stroke is more common than hemorrhagic stroke and reported the overall mortality associated with stroke. However, none of these studies assessed the in-hospital morality associated with ischemic stroke and the studies suffer from small sample size. The aim of this study was to determine the rate and predictors of in-hospital mortality due to ischemic stroke in Gondar University Hospital (GUH).

## 2. Materials and Methods

### 2.1. Study Design and Setting

The current study was conducted from April 1, 2017, to May 15, 2017. It was based on a similar study design and setting done by Gebreyohannes et al. [11] on medical records of patients with atrial fibrillation during the same period of time. However, the current study was on different study population (i.e., hospitalized ischemic stroke patients) with different study objectives and sample sizes. Medical records of patients, 18 years and older, with the diagnosis of hyperacute and acute ischemic stroke were included in the present study.

### 2.2. Outcome Measures

The primary endpoint was in-hospital mortality. Secondary endpoints include length of hospital stay.

### 2.3. Statistical Analysis

Descriptive statistics were used to summarize sociodemographic and other information. Categorical variables were expressed as frequencies (percentage) and quantitative variables as mean ± standard deviation or median + interquartile range (IQR)/range. Prior to additional analyses, Shapiro–Wilk and Levene test was performed to assess the data for normality and homogeneity. Cox hazard regression was used to determine the predictors of in-hospital mortality by including into the model all the candidate variables (variables with* p*≤0.10 in bivariate, except those with a high number of missing data). Independent-sample t-test and Pearson's correlation were used to identify factors associated with duration of hospital stay. A two-sided statistical test at 5% level of significance was used. All of the analyses were performed using statistical package for social sciences (SPSS) version 20 (IBM Corp., Armonk, NY).

### 2.4. Definition of Terms and Operational Definitions

TOAST classification was used to categorize ischemic stroke into five subtypes: cardioembolic (CE), large artery atherosclerosis (LAA), small vessel occlusion (SVO), other determined etiology (ODE), and undetermined etiology (UDE). UDE was used to indicate any of the following: two or more causes identified, negative evaluation, or incomplete evaluation [12].

Hyperacute ischemic stroke is ischemic stroke with symptom onset-to-arrival time within 24 hours; acute ischemic stroke is ischemic stroke with symptom onset-to-arrival time within 24 hours to seven days [13].

### 2.5. Ethics Approval

Ethical clearance was obtained from the Ethical Clearance Committee of School of Pharmacy, College of Medicine and Health Sciences (CMHS), University of Gondar (UOG). Confidentiality of the information regarding patients was ensured in such a way that the data will only be used for the study purpose only. Also, the information obtained from the patients' medical records is presented only in collective manner.

## 3. Results

### 3.1. Patients' Characteristics

A total of 229 medical records of patients with ischemic stroke were identified. Of these, 21 patient medical records were either incomplete, lost, patients came after a week, or contained medical records where patients went against medical advice. The final analysis included 208 ischemic stroke patients ranging from 18 to 88 years of age. The mean (median) age of the patients was 65.17 (68) years with female majority (57.5%). The mean symptom onset-to-arrival time was 60.42 hours. There was a wide gap in symptom onset-to-arrival time: from 1 hour to 168 hours. Hypertension was the most common risk factor (N=118), followed by AF (N=76) and CHF (N=70) (Table 1). Selected laboratory investigations done at admission are shown in Table 2.

The mean (±SD) duration of hospital stay was 11.55 (10.040) days. Of the total 208 patients, 26 (12.5%) patients died in the hospital. Potential causes of in-hospital mortality among these patients are presented in the Supplementary Material section. These patients had different potential causes of death. Of these, 25 patients had hemiplegia or hemiparesis (24 uncrossed vs. 1 crossed, and 18 hemiplegia vs. 7 hemiparesis), 15 patients developed one or more infections (12 aspiration pneumonia, 2 hospital-acquired pneumonia, 1 urinary tract infection, and 1 bed-sore), and 2 patients had hemorrhagic transformations (Supplementary Material (available here)). Data on 3-month survival was available for only 84 patients and there was no other mortality other than those who died in the hospital. Nearly one-third (29.33%) of the patients experienced aphasia. Of these, the majority of the patients experienced Broca's aphasia (52) and the rest experienced Global (7) and Wernike's (2) aphasia. Most patients experienced body weakness, the most common of which was hemiplegia (N=112) followed by hemiparesis (N=78). Twelve patients experienced hemorrhagic transformation (Table 3).

**Figure 1:**
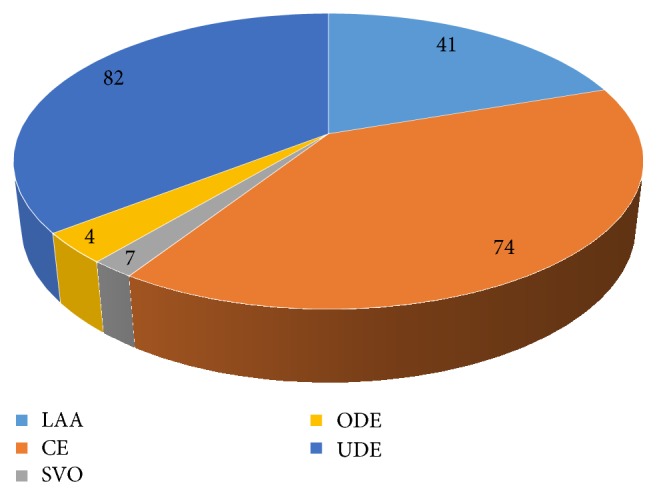
Cause of ischemic stroke.

Most patients had UDE (N=82). CE was the most common identified cause of ischemic stroke (N=74) (Figure 1). Seven patients had ODE; of these, 3 had brain atrophy while the remaining one had TB meningitis.

**Figure 2:**
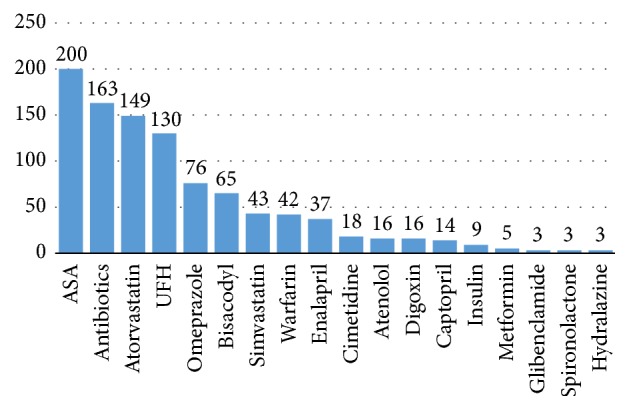
Most commonly prescribed medications: ASA: aspirin and UFH: unfractionated heparin.

ASA was the most commonly prescribed medication followed by lipid-lowering agents. Atorvastatin and simvastatin were the only lipid-lowering agents used by the patients with atorvastatin being prescribed more frequently. Other commonly prescribed medications include antibiotics, UFH, omeprazole, and bisacodyl (Figure 2).

Binary logistic regression failed to reveal any association between age, sex, GCS on admission, headache, vomiting, seizure, and aphasia, with early (<3 hours) hospital arrival. Upon bivariate analysis, Cox proportional hazard regression revealed that the presence of any infection and an increase in serum creatinine were associated with higher risk of in-hospital mortality in patients with ischemic stroke. On the other hand, the presence of vascular disease, any type of VHD, a higher GCS score on admission, and use of statins were associated with a decrease in the risk of in-hospital mortality among ischemic stroke patients. However, upon multivariate analysis, only a decrease in renal function, particularly elevated serum creatinine (AHR=8.848, 95% CI: 1.616-67.437) was associated with a statistically significant increase in-hospital mortality (Table 4).

Independent-sample t-test revealed that male sex, absence of any type of VHD shortened the duration of hospital stay. On the other hand, use of lipid-lowering agents, warfarin, and DVT prophylaxis prolonged length of hospital stay. Pearson's correlation showed that hemoglobin, SGOT, SGPT, RBS, and SBP had weak relationship with duration of hospital stay (Table 5).

## 4. Discussion

This retrospective analysis of medical records assessed the rate and predictors of in-hospital mortality in patients admitted due to ischemic stroke. The findings of this study showed a 12.5% rate of in-hospital mortality due to ischemic stroke. This is more than double overall mortality in Germany that was reported by Heuschmann et al. [14]; however, the mortality rate in the Heuschmann study varied from hospital to hospital and ranged from 0 to 25%. On the other hand, a study conducted in Ghana reported a higher mortality rate (17.5%) from ischemic stroke than the present study [15]. While several factors were identified as predictors of in-hospital mortality upon bivariate analysis, serum creatinine and BUN were the only factors that independently predicted in-hospital mortality. In particular a rise in serum creatinine showed more than eightfold increase in in-hospital mortality.

The mean (median) duration of hospital stay was 11.55 (9.0) days with a wide variation ranging from just 1 day to 71 days. This was close to the mean length of hospital stay of 10.6 days which was reported by Heuschmann et al. [14]. In the present study, male sex, absence of any type of VHD shortened the duration of hospital stay. On the other hand, use of lipid-lowering agents, warfarin, and DVT prophylaxis prolonged length of hospital stay.

The principal aim of the stroke care is to reduce the mortality and long-term disability. The success of stroke therapy depends on its early management. Several studies have evaluated the importance of symptom onset-to-arrival time and its implications in-hospital outcomes [16–20]. It is evident that early treatment of acute ischemia reduces stroke complications, reduces mortality, and shortens hospital stay [21–23]. In the current study, the symptom onset-to-arrival time varied considerably from within 1 hour to 168 hours. The median symptom onset-to-arrival time was 24 hours which was much longer than the 1.5 hour reported by Agyeman et al. [24]. Just below half of the patients seek medical attention after 48 hours and only 3.2% of them came to the hospital within 3 hours. This indicates patients' tendency to seek medical attention late. Previous studies identified male sex [25] and atrial fibrillation [26] as predictors of earlier arrival to the hospital, but these were not identified as predictors in the present study. The small sample size could have contributed to the inability of the present study to identify these factors as predictors. Kim et al. reported that awareness of the initial symptom was a stroke by the patient/bystander and knowledge about thrombolysis was associated with early arrival to the hospital [26]. As the prognosis of ischemic stroke gets poorer, in terms of mortality and disability, with a delay to seek medical attention, much work should be done to improve knowledge and attitude towards stroke treatment and the importance of early intervention on patient outcomes. However, despite the wide variability in symptom onset-to-admission time, it did not show statistical significant association with in-hospital mortality.

Intravenous thrombolysis with recombinant tissue plasminogen activator (rtPA) remains corner stone to improve patient outcomes in acute ischemic stroke [16]. Thrombolytic agents aid clot dissolution and timely restoration of the blood flow to the ischemic brain tissue and thus prevent death of neurons and lead to clinical improvement. Twenty-nine patients arrived in the hospital within 4.5 hours; however, due to lack of rtPA in our setting, almost all patients were given only ASA and that may contribute to poorer outcomes. Long time intervals between onset of symptoms to admission and lack of stroke unit or emergency medical services for aggressive management still remained major barriers in our setting.

In the study, hypertension is identified as the most prevalent modifiable risk factor and is also a powerful modifiable risk factor for small vessels which can attribute risk between 35-50% [27]. Studies highlighted that nearly 90% of strokes occur among patients with resistant hypertension [28] and identifying the underlying cause and treating them specially can significantly prevent the stroke risk. In particular to our population, identifying the hereditary cause of hypertension and their extent of salt and water retention can distinguish their type of hypertension and this can facilitate to tailor necessary interventions to control their blood pressure. Some of such interventions were also discussed in previous literatures [29, 30]. In addition, atrial fibrillation was also identified in more than one-third of the ischemic stroke patients. It is evident from the previous studies that atrial fibrillation or flutter is known to cause stroke in about 20% of the patients presenting with acute ischemic stroke. The American Heart Association recommends starting all patients with stroke and atrial fibrillation on anticoagulation, unless no contraindication is recommended [31]. However, the benefit and timing of therapeutic anticoagulation in ischemic stroke patients remain controversial.

The etiologies of ischemic stroke are diverse and differences in operating definitions exist among stroke subtypes. The current study used TOAST [12], a first classification system based on stroke mechanism in our study and still most widely used. According to this classification, ischemic stroke patients are classified into five subtypes. The original TOAST study reported that outcomes and recurrence differ depending on the ischemic stroke subtype. However, the current study did not identify any association between the ischemic stroke subtype and in-hospital mortality.

Interpretation of the current study should be done by taking following limitations into consideration. It was a retrospective study design and suffered from incompleteness and even loss of patients' medical records. The sample size was small which may obscure the effect of some potential predictors that would have been identified with a larger sample size. The study also was unable to adequately assess 3-month mortality due to high rate of loss to follow-up.

## 5. Conclusion

The in-hospital mortality associated with ischemic stroke was found to be high. Mainly, elevation in serum creatinine was highly associated with poorer outcomes in terms of in-hospital mortality. Duration of hospital stay was not significantly affected by any factors and was comparable to other studies. Patients' tendency to seek medical attention early was found to be poor and could contribute to poor outcomes. Much work should be done on improving the knowledge and awareness of the community regarding ischemic stroke and stroke in general to encourage early medical seeking behavior and reduce mortality and long-term disability.

## Figures and Tables

**Table 1:** Sociodemographic and clinical characteristics of ischemic stroke patients.

**Characteristics**	
Age	

Mean (±SD)	65.17 (14.068)

Median (IQR)	68 (18)

Sex N (%)	

Male	88 (42.3%)

Female	120 (57.7%)

Mean symptom onset-to-arrival time in hours (±SD)	60.420 (58.552)

Mean SBP at admission (±SD)	138.63 (30.016) mm Hg

Mean DBP at admission(±SD)	82.30 (16.079) mm Hg

Mean PR at admission (±SD)	87.25 (15.726) beats/min

Mean RR at admission (±SD)	22.48 (4.317) breath/min

Mean body temperature at admission (±SD)	38.3092 (23.7405)°C

Mean GCS at admission (±SD)	12.33 (2.758)

Risk factor N (%)	

AF	76 (36.5)

DM	13 (6.3)

CAD/IHD	14 (6.7)

CHF	70 (33.7)

Previous MI	5 (2.4)

Vascular disease	61 (29.3)

Hypertension	118 (56.7)

Any VHD	72 (34.6)

**Table 2:** Laboratory results of ischemic stroke patients on admission.

Investigations	Mean (±SD)
Hemoglobin (N=176)	14.6897 (9.97) mg/dL

Hematocrit (N=185)	41.5638% (6.379)

SGOT (AST) (N=160)	38.098 (31.359) units/L

SGPT (ALT) (N=159)	29.182 (32.706) units/L

BUN (N=136)	31.807 (22.171) mg/dL

Serum creatinine (N=170)	0.9456 (0.4019) mg/dL

RBS (N=174)	135.033 (48.245) mg/dL

**Table 3:** Complications experienced by the study participant due to ischemic stroke.

**Complications**	**N (**%**)**
Presence of aphasia	61 (29.33%)

Paresis	82 (39.42%)

Hemiparesis	78 (37.50%)

Monoparesis	2 (0.96%)

Quadriparesis	2 (0.96%)

Plegia	113 (54.33%)

Hemiplegia	112 (53.85%)

Monoplegia	1 (0.48%)

Any infection	78 (37.5%)

Hemorrhagic transformation	12 (5.77%)

In-hospital death	26 (12.5%)

**Table 4:** Factors that predict in-hospital mortality among ischemic stroke patients.

**Factors**	**CHR (95**%** CI)**	***P* value**	**AHR (95**%** CI)**	***P* value**
Vascular disease	0.186 (0.044-0.791)	0.023	1.176 (0.163-8.489)	0.872

Any VHD	0.264 (0.090-0.772)	0.015	0.174 (0.016-1.863)	0.148

Any infection	6.341 (2.389-16.832)	<0.001	1.066 (0.178-6.388)	0.944

Hematocrit	0.950 (0.897-1.006)	0.080	0.997 (0.907-1.097)	0.957

SGOT	1.010 (1.001-1.019)	0.025	1.023 (0.989-1.057)	0.191

BUN	1.020 (1.009-1.030)	<0.001	1.020 (1.003-1.037)	**0.019**

Serum creatinine	5.002 (2.217-11.285)	<0.001	8.848 (1.616-67.437)	**0.035**

GCS on admission	0.805 (0.708-0.916)	0.001	0.938 (0.706-1.246)	0.660

Statin use	0.309 (0.105-0.916)	0.034	0.670 (0.100-4.480)	0.679

Warfarin use	0.265 (0.062-1.125)	0.072	1.393 (0.127-15.311)	0.787

Hemorrhagic transformation	9.805 (4.001-24.029)	<0.001	1.029 (0.118-12.399)	0.873

**Table 5:** Factors affecting duration of hospital stay in patients with ischemic stroke.

**Independent sample t-test**		

**Factors**	**Mean duration of hospital stay (days)**	***P* value**

Sex		**0.048**

Male	9.94	

Female	12.73	

Any VHD		**0.002**

No	9.71	

Yes	14.99	

Lipid-lowering agents		**0.029**

No	6.47	

Yes	12.00	

Warfarin		**0.035**

No	10.81	

Yes	14.45	

DVT prophylaxis		**0.013**

No	9.33	

Yes	12.88	

**Pearson correlation**		

**Factors**	**r (df)**	***P* value**

Hemoglobin	0.221 (174)	0.030

SGOT (AST)	0.159 (157)	0.046

SGPT (ALT)	0.287 (156)	<0.001

RBS	0.249 (171)	0.001

SBP	-0.142 (205)	0.041

df: degree of freedom.

## Data Availability

All data generated or analyzed during this study are included in this published article.

## Abbreviations

AF: Atrial Fibrillation
AHR: Adjusted Hazard Ratio
ALT: Alanine Transaminase
AOR: Adjusted Odds Ratio
AST: Aspartate Transaminase
BUN: Blood Urea Nitrogen
CE: Cardioembolic
CAD: Coronary Artery Disease
CHF: Congestive Heart Failure
CI: Confidence Interval
CMHS: College of Medicine and Health Sciences
CHR: Crude Hazard Ratio
DM: Diabetes Mellitus
DVT: Deep Vein Thrombosis
ESR: Erythrocyte Sedimentation Rate
GCS: Glasgow Coma Scale
GUH: Gondar University Hospital
HDL: High-Density Lipoprotein
IHD: Ischemic Heart Disease
IQR: Interquartile Range
LAA: Large artery atherosclerosis
LDL: Low-Density Lipoprotein
LVEF: Left Ventricular Ejection Fraction
ODE: Other determined etiology
RBS: Random Blood Sugar
rtPA: Recombinant Tissue Plasminogen Activator
SD: Standard Deviation
SGOT: Serum Glutamic Oxaloacetic Transaminase
SGPT: Serum Glutamic-Pyruviv Transaminase
SPSS: Statistical Package for Social Sciences
SVO: Small vessel occlusion
UDE: Undetermined etiology (UDE)
UFH: Unfractionated Heparin
UOG: University of Gondar
VHD: Valvular Heart Disease.

## Supplementary Materials

Supplementary Materials Supplementary data include potential causes of in-hospital mortality among ischemic stroke patients. These include infections, hemorrhagic transformations, cardiac conditions, coma, and others.
