# Supplementary material for: In-Hospital Mortality among Ischemic Stroke Patients in Gondar University Hospital: A Retrospective Cohort Study
Source: Stroke Res Treat. 2019 Jan 1;2019:7275063. doi: 10.1155/2019/7275063 (PMC6332873; doi:10.1155/2019/7275063)
Supplement: Supplementary Materials — Supplementary data include potential causes of in-hospital mortality among ischemic stroke patients. These include infections, hemorrhagic transformations, cardiac conditions, coma, and others. [file 7275063.f1.pdf]

Supplementary material: Potential causes of in-hospital mortality among ischemic stroke patients

Potential causes of in-hospital mortality	Number of patients (N=26)
Uncrossed hemiplegia secondary to IS + aspiration pneumonia	4
Uncrossed hemiplegia secondary to CE IS + AF	3
Uncrossed hemiplegia secondary to IS + grade IV bed sore	1
Uncrossed hemiplegia + aspiration pneumonia + HAP	1
Uncrossed hemiplegia secondary to IS + Broca’s aphasia	1
Uncrossed hemiplegia secondary to IS + Global aphasia	1
Uncrossed hemiplegia secondary to CE IS + AF + aspiration pneumonia + Killip’s class III ACS	1
Uncrossed hemiplegia secondary to CE IS	1
Uncrossed hemiplegia secondary to IS with hemorrhagic transformation	1
Uncrossed hemiparesis secondary to CE IS with hemorrhagic transformation + AF+ aspiration pneumonia	1
Uncrossed hemiparesis secondary to IS + Broca’s aphasia + aspiration pneumonia	1
Uncrossed hemiparesis secondary to IS	1
Uncrossed hemiparesis secondary to IS + aspiration pneumonia	1
Uncrossed hemiparesis secondary to IS + Broca’s aphasia	1
Uncrossed spastic hemiparesis secondary to IS + Broca’s aphasia + aspiration pneumonia	1
Uncrossed hemiplegia secondary to IS + NYHA class IV stage C CHF secondary to core pulmonale + COPD + aspiration pneumonia	1
Coma secondary to IS + aspiration pneumonia + HAP	1
Coma secondary to Uncrossed hemiplegia secondary to IS	1
Coma secondary to crossed flaccid left sided hemiplegia secondary to CE IS	1
Coma secondary to Uncrossed hemiplegia secondary to CE IS + AF + aspiration pneumonia	1
Coma secondary to Uncrossed hemiparesis secondary to IS + Broca’s aphasia + aspiration pneumonia	1

Note: ACS: Acute Coronary Syndrome, AF: Atrial Fibrillation, CE: Cardioembolic, CHF: Congestive Heart Failure, COPD: Chronic Obstructive Pulmonary Disease, HAP: Hospital-Acquired Pneumonia, IS: Ischemic Stroke, NYHA: New York Heart Association, UTI: Urinary Tract Infection,
